# Improving social resilience amid the COVID-19 epidemic: A system dynamics model

**DOI:** 10.1371/journal.pone.0294108

**Published:** 2023-11-09

**Authors:** Chenhuan Kou, Xiuli Yang

**Affiliations:** 1 College of Economics & Management, Heilongjiang Bayi Agricultural University, Daqing, Heilongjiang, China; 2 School of Public Administration and Law, Northeast Agricultural University, Harbin, Heilongjiang, China; HUTECH University, VIET NAM

## Abstract

Social resilience is a key factor in disaster management, but compared to resilience in other fields, research on social resilience is still limited to assessment or evaluation, and there is still a lack of dynamic and procedural research, which is also a challenge. This article constructs a causal feedback model and a system dynamics model of social resilience during the COVID-19 epidemic, so as to analyze the dynamic characteristics and improvement path of social resilience. After verifying the effectiveness of the model, model simulation is conducted and the following important conclusions are drawn: social resilience dynamically changes during the research cycle and is influenced by social entity behavior and social mechanisms; The sensitivity factors for the two variables that measure social resilience, namely panic degree and damage degree, are the real-time information acquisition of public and the epidemic awareness of local government, respectively. Therefore, the path to enhancing social resilience should be pursued from both the public and government perspectives, including improving the public’s ability to access real-time information, increasing the timeline of government information disclosure, and enhancing local governments’ understanding and awareness of the epidemic.

## Introduction

Cities have been increasingly exposed to various risks and crises [1], such as global climate change, financial crisis, disease, epidemic, etc. The COVID-19 epidemic that broke out in early 2020 has had a serious and far-reaching impact on the city. By May 2021, more than 188 countries and regions have reported over 160 million infected people and more than 3.32 million deaths, which has triggered the global economic recession [2]. The emphasis on resilience in advance defense provides new ideas for cities to achieve sustainable development in times of crisis [3–5]. Resilience has become a leading idea and has been valued by many international organizations. For example, in 2003, Rockefeller Foundation launched the “Global 100 Resilient Cities” project to enhance urban resilience to meet future challenges. In 2005, the Second World Conference on Disaster Reduction, held in Hyogo County, Japan, listed resilience as the focus of discussion.

Social resilience is the application of resilience thinking in the social-ecological system, which has been highlighted as a key factor in disaster management [6]. Social resilience is the mapping of social entities, social mechanisms, and their interrelationships, manifested as resistance to risks and rapid recovery and reconstruction. Therefore, social resilience is not a static attribute, it can undergo dynamic evolution, but research on the dynamics and processes of social resilience is still relatively lacking.

In view of this, this article will study the dynamic characteristics and improvement path of social resilience by building a system dynamics model against the background of the COVID-19 epidemic. The system dynamics model is based on the causal feedback mechanism and can be well applied to depict the behavior of social entities and social mechanisms. The study of social resilience using system dynamics is a new perspective.

The remainder of this article is as follows: Section 2 is the literature review, which elaborates on the definition and evolution of resilience, as well as the definition and research status of social resilience; Section 3 is the methodology, which introduces the research methods used in this article and their applicability and rationality. Section 4 is the model development, which describes the model structure and variable setting, casual feedback model, and system dynamics model; Section 5 is model simulation, which details data resource and model set, model testing, simulation analysis, and scenario analysis; Section 6 is discussion and conclusion, which provides discussion, conclusion, and research limitations.

## Literature review

This section reviews the definition and evolution of resilience and the definition and research status of social resilience. Based on the literature review, this article elaborates on the research innovations.

### Definition and evolution of resilience

The term “resilience” originates from the Latin word “resilio” and is used to describe a spring back or bounce back. In academic study resilience first appeared in the research field of classical physics, mainly referring to the speed at which a spring rebounds back to its initial size and shape after deformation. With the continuous advancement of industrialization, resilience has been introduced into the research field of mechanics to describe the ability of materials to recover to their original form after being subjected to external force. In 1973, Holling, an ecology professor at the University of Florida in the United States, introduced resilience thinking into the areas of natural ecology and human ecology, realizing the expansion of the concept and connotation of resilience [7]. In summary, the concept of resilience has experienced at least three extensions, from engineering resilience in the field of mechanics, ecological resilience in the field of natural ecology, and evolutionary resilience in the field of human ecology.

#### Engineering resilience

Engineering resilience originated in the field of mechanics and is used to describe the ability to bounce back of materials after deformation. Engineering resilience is closest to people’s understanding of resilience in their daily lives. This article lists some scholars’ definitions of the concept of engineering resilience. For example, Holling (1973) believes that engineering resilience refers to the ability of a system to quickly recover after deviating from a steady state [8]. Berkes & Folk (1998) pointed out that engineering resilience emphasizes the ability of the system to maintain stability or recover quickly after deviating from the equilibrium state, so it can be measured by the time when the system recovers to the equilibrium state after being disturbed [9].

From the perspective of the aforementioned scholars, it can be seen that engineering resilience emphasizes equilibrium and stability and believes that there is only one stable state in the system. Engineering resilience refers to the ability of a system to maintain stability or quickly recover stability after being disturbed.

#### Ecological resilience

With a deepening understanding of the internal structural characteristics of the system, scholars have found that the system can have multiple steady states. Therefore, after being disturbed, the system may either return to the initial steady state or transition to another steady state, and the disturbance is the opportunity for steady-state transition. In this situation, the recognition of engineering resilience is difficult to reflect the true behavioral characteristics of the system.

Ecological resilience is an extension of the concept of engineering resilience. Holling distinguished the meanings of the two in *Engineering Resilience Versus Ecological Resilience*. Ecological resilience emphasizes the evolution and change of a system [10], believing that there may be multiple steady states that resilience should be measured by the maximum level of disturbance that the system can withstand before transitioning to a new steady state.

Compared to engineering resilience, the focus of ecological resilience has shifted from behavioral constancy to systemic structural sustainability. Therefore, ecological resilience refers to the ability of a system to return to its initial steady state or transition to another steady state after experiencing external disturbances.

#### Evolutionary resilience

Complex system theory suggests that a complex adaptive system may not have a steady state but is constantly evolving dynamically. This viewpoint questions the premise of engineering resilience and ecological resilience, and once again expands the concept of resilience, resulting in evolutionary resilience. During this period, an important model emerged: The adaptive cycle. This model divides the dynamic evolution of the system into four nested stages, namely: Exploration phase, conservation phase, release phase, and reorganization phase. Resilience dynamically changes during these four stages.

Evolutionary resilience is a new recognition of concept, which breaks the long-term pursuit of stability in resilience research and emphasizes the ability of adaptability, learning, innovation, and transformation of the system. Therefore, under the premise of the complex evolution of the social-ecological system, evolutionary resilience should not only be explained as the ability of the system to maintain and restore the stable state, but also as the ability of the system to resist, adapt, and transform to respond to pressure [11].

This article compares three types of concept cognition of resilience, as shown in Table 1.

**Table 1:** Comparison of three e types of concept cognition of resilience.

Concept cognition	Focus	Feature	Research field	Definition
Engineering resilience	Unique steady state	Recovery	Mechanics	The time and speed at which the system recovers to its unique steady state after being disturbed.
		Constant		
Ecological resilience	Multiple steady state	Buffer	Natural ecology	The maximum disturbance level that the system can absorb before crossing the threshold and transitioning to another steady state.
		Resistance		
		Maintenance		
Evolutionary resilience	No steady state	Adaptation	Human ecology	The ability of the system to resist disturbances and maintain normal functions, as well as the ability to adapt, self-organize, learn, and innovate.
	Multi-scale	Restructure		
	adaptive cycle	Transformation		

### Definition and research status of social resilience

#### Definition of social resilience

Social resilience is the specific application of resilience thinking in the social-ecological field, which is often recognized as a critical dimension of resilience. For example, Bruneau et al. (2003) developed a TOSE analysis framework of resilience consisting of four interrelated sub-dimensions: technical resilience, organizational resilience, social resilience, and economic resilience [12]. Burton (2015) constructed an evaluation index system for resilience, covering four sub-dimensions: social resilience, economic resilience, governance resilience, and infrastructural resilience [13].

Overall, the definition of social resilience in existing research can be divided into three categories: the ability of social entities, the ability of social mechanisms, the ability of social entities and mechanisms. Social entities mainly include families, communities, social organizations, social groups, etc., while social mechanisms refer to the ability to manage risks, self-organization, and change.

The first type of definition focuses on the ability of social entities to respond to or recover from risks. For example, Bruneau et al. (2003) proposed that social resilience is the ability of social units (communities, social organizations, etc.) to mitigate the impact of disasters and reduce social losses and chaos [12]. Maguire and Hagan (2007) defined social resilience as the ability of social groups and communities to actively respond to and recover from crises [14]. Khalili et al. (2015) defined social resilience as the ability of communities to reduce losses and rebuild in disasters [15].

The second type of definition focuses on the ability of social mechanisms to cope, adapt, and transform. For example, Rockström (2003) argues that social resilience is a social coping mechanism used to cope with extremely uncontrollable impacts [16]. Shaw (2014) stated that social resilience is determined by risk perception, accepting change, and self organization, which can be expressed as “social resilience = risk perception × self perception × accepting change × self organization” [17].

The third type of definition is a combination of the first two, which considers both the risk resistance of social entities and the adaptability and transformation ability of social mechanisms. For example, Kwok et al. (2016) defined social resilience as the ability of a community’s social environment to effectively anticipate, respond to, and recover from disasters [6].

**Fig 1:**
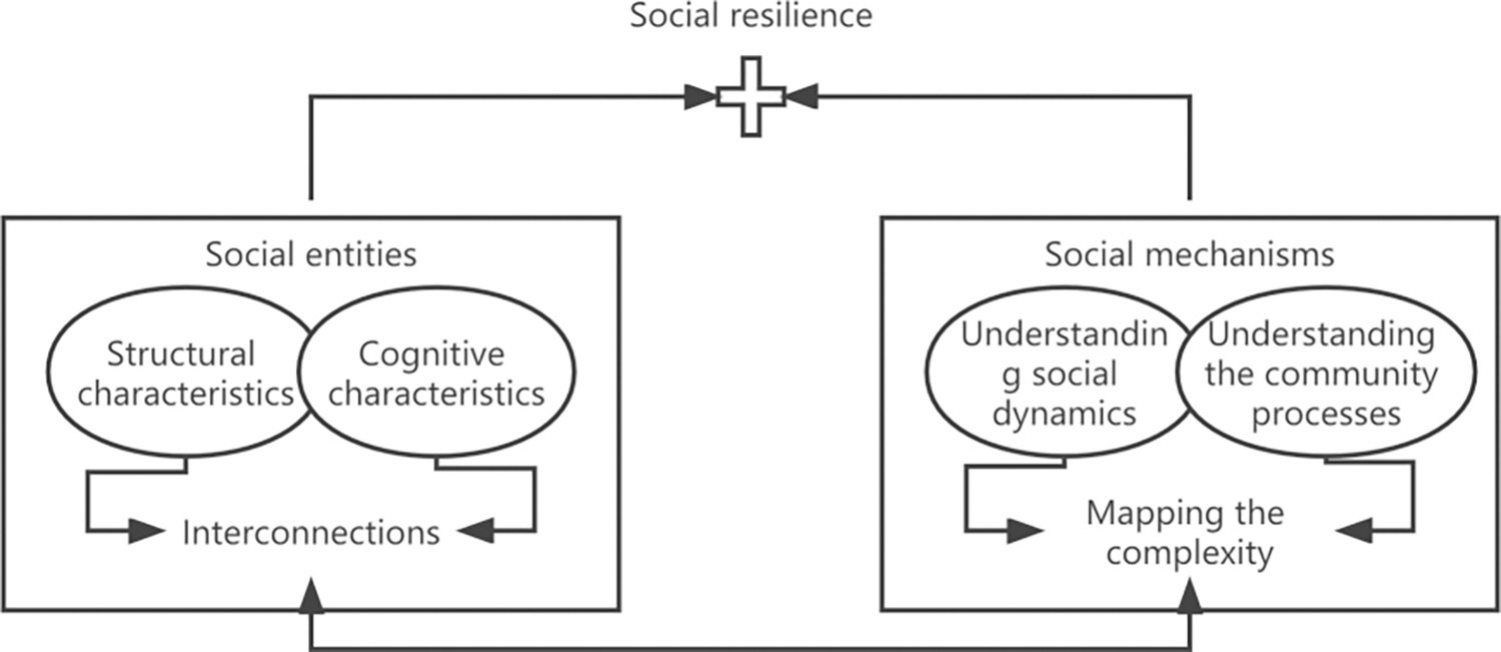
Social resilience is defined as abilities of social entities and mechanisms [18].

In the process of coping with shocks, social entities that withstand internal and external environmental pressures are essentially complex adaptive systems (CAS). The concept of social resilience should include the mapping of the capabilities of social entities and mechanisms and their interrelationships, as shown in Fig 1.

Therefore, this article adopts the following definition of social resilience, which is social resilience refers to the ability of social entities and social mechanisms to effectively resist, mitigate, and cope with disasters and quickly recover to minimize social disruptions [19].

#### Principal characteristic of social resilience

Existing research has analyzed and summarized the characteristics of social resilience from multiple aspects, which can be summarized into three aspects [20–23]: resistance, adaptability, and transformability. This is very similar to the main characteristics of evolutionary resilience, as these terms are referred to by the Resilience Alliance, an interdisciplinary association that studies resilience.

The first characteristic is resistance, commonly referred to as persistence [8, 11, 24]. This characteristic is used to describe the degree to which complex systems can withstand changes or transformations while maintaining functional and structural characteristics. In terms of social resilience, resistance not only refers to the ability of the social system to maintain stability but also implies its ability to resist shocks before reaching functional and structural collapse.

The second characteristic is adaptability, which mainly emphasizes the ability of the social system to absorb changes. That is the ability of agents to learn from experience, accumulate knowledge, and respond quickly to constantly changing environments [25–28]. Therefore, the adaptability of social resilience describes the self-organizing and restructuring ability of the social system under environmental pressure [29–31], highlighting the dynamic and evolutionary nature of resilience.

The third characteristic is transformability, which refers to the ability of the social system to transition to a new state. Transformability implies the possibility of evolution in the social system, and shocks or disturbances are opportunities for transformation. Therefore, the social system can respond to challenges through continuous learning [9, 28], thereby maintaining survival and sustainability.

**Fig 2:**
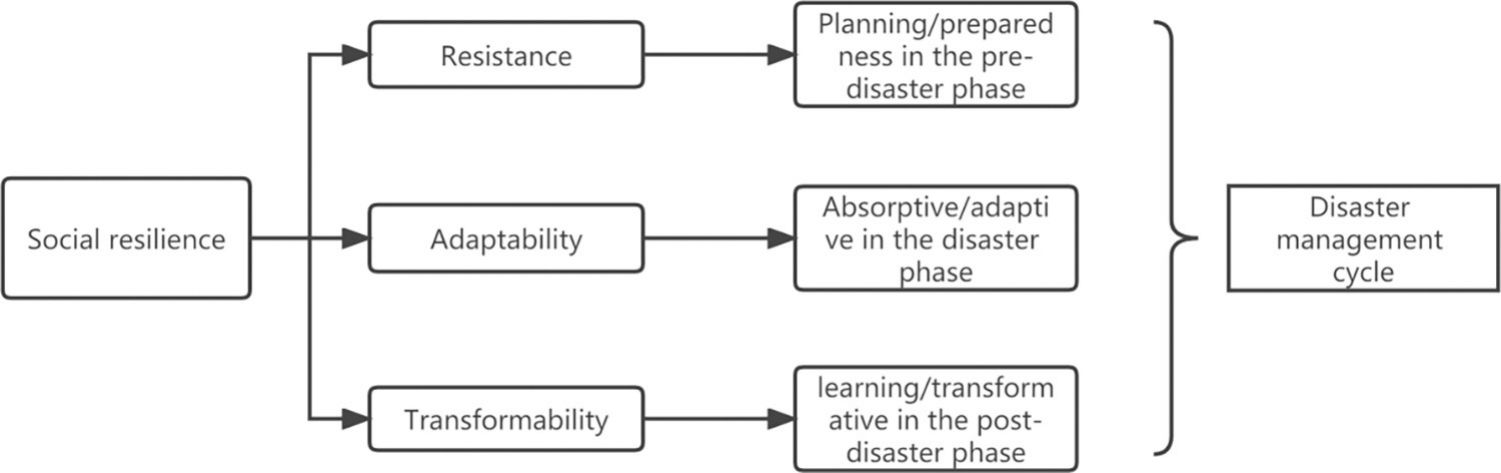
Three characteristics of social resilience correspond to the disaster management cycle.

The above three characteristics of social resilience describe the ability of the social system to relieve stress (resistance), absorb change (adaptability), and evolve and transform (transformability). These characteristics also demonstrate that social resilience is a dynamic process, not just a stable state. Moreover, these three characteristics correspond to the complete cycle of disaster management, namely: planning/preparedness in the pre-disaster phase, absorptive/adaptive in the disaster phase, and learning/transformative in the post-disaster phase, as shown in Fig 2.

#### Quantitative research of social resilience

A key challenge in quantitative research on social resilience is how to operationalize abstract concepts so that they can be measured, compared, and dynamically analyzed. At present, quantitative research on social resilience mainly focuses on its assessment or measurement, and the commonly used research method is to construct a framework covering a series of indicators based on the characteristics of social resilience. Research data is mostly sourced from publicly available census data [32, 33]. For example, the number of citizen organizations is used to represent the degree of citizen participation in social networks [34], and the years of education are used to measure adaptability [35]. Representative studies include: Saja et al. (2018) constructed a “5S” assessment framework for social resilience by selecting 46 indicators from five sub-dimensions (structure, capital, mechanism, equity, and belief) [19]. Sharifi (2016) constructed the assessment framework for social resilience from five dimensions: social structure, social capital, safety and well-being, equity and diversity, and local culture [36]. Some scholars have also used interviews or surveys to assess the level of social resilience, such as research by Ainuddin et al. (2015) [37] and Joerin et al. (2014) [38].

In the critical review by Saja et al. (2019), 31 measurement frameworks for social resilience were reviewed in 172 literature, which involved over 80 indicators, including human capital, lifestyle, community competence, community capital, demographics, and risk knowledge [18]. At the same time, Saja et al. (2019) pointed out in the critical review that there are still three deficiencies in the existing framework for measuring social resilience. One is that many measurement frameworks have not been implemented. Secondly, existing measurement frameworks overlook dynamic, process-oriented indicators. Thirdly, using census data to select measurement indicators has strong limitations and may not have objective or qualitative analysis indicators.

Although social resilience is not only an outcome but also a process-oriented phenomenon that has become a consensus [39], there is still a significant lack of dynamic research on social resilience. This is also the main innovation of this article, which is to construct a dynamic simulation model of social resilience, depict the dynamic development of social resilience, and explore the determinants of enhancing social resilience.

### Research innovation

The current research status on social resilience indicates that social resilience has obvious dynamic characteristics, and its evolution process is not limited to a stable state. Conducting dynamic and process analysis of social resilience is the current key point and difficulty in this field. Therefore, this article will take the COVID-19 epidemic as an example to study the dynamic changes and improvement measures of social resilience through building models and model simulation.

**Fig 3:**
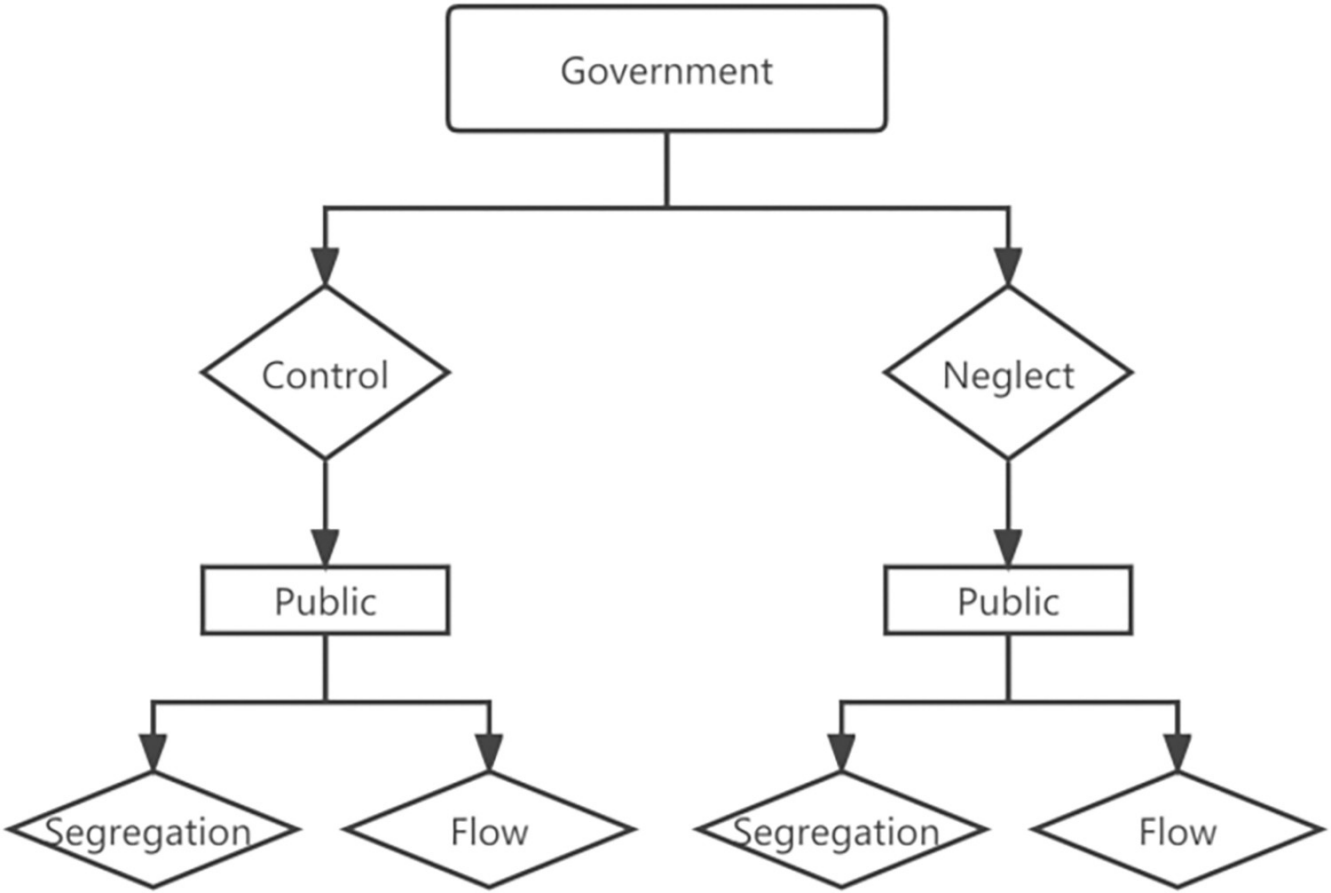
The game behavior between government and public.

During the epidemic, there is a game behavior between the government and the public, as shown in Fig 3.

Fig 3 shows that for residents who have been infected or have a high probability of infection, the government will adopt two behavioral strategies, namely control and neglect, with a strategy set of *S*_1_ = {*C, N*}. Among them, control refers to the government taking proactive measures such as regulation and treatment to block the virus transmission chain. Neglecting refers to the lack of proactive measures taken by the government to reduce the spread of the virus. According to the government’s behavior, residents who have been infected or have a high probability of infection also have two corresponding behavioral strategies, namely segregation and flow, with a set of strategies as *S*_2_ = {*S, F*}. Segregation includes home quarantine and centralized quarantine, both of which refer to residents voluntarily reducing the frequency of social activities to avoid actively spreading the virus. Flow refers to residents following a conventional life trajectory and is prone to increasing the spread of the virus and causing more people to be infected during public transportation or interpersonal communication.

For the government, choosing a neglected behavioral strategy will lead to a rapid increase in the number of infections and the collapse of the healthcare system, thereby reducing social stability. But if too strict regulatory measures are taken, it can easily affect economic development. For the public, the choice of segregation or flow behavior strategy depends more on whether the government’s regulatory measures are strict.

From this, it can be seen that the entire game process has obvious causality and feedback. Therefore, this article will adopt a system dynamics (SD) approach to construct a research model.

## Methodology

Social resilience can be conceptualized as both an outcome and a process, and process-oriented characteristics require more effort in systems thinking. This article constructs system dynamics (SD) model to study the dynamic evolution and improvement path of social resilience.

**Fig 4:**
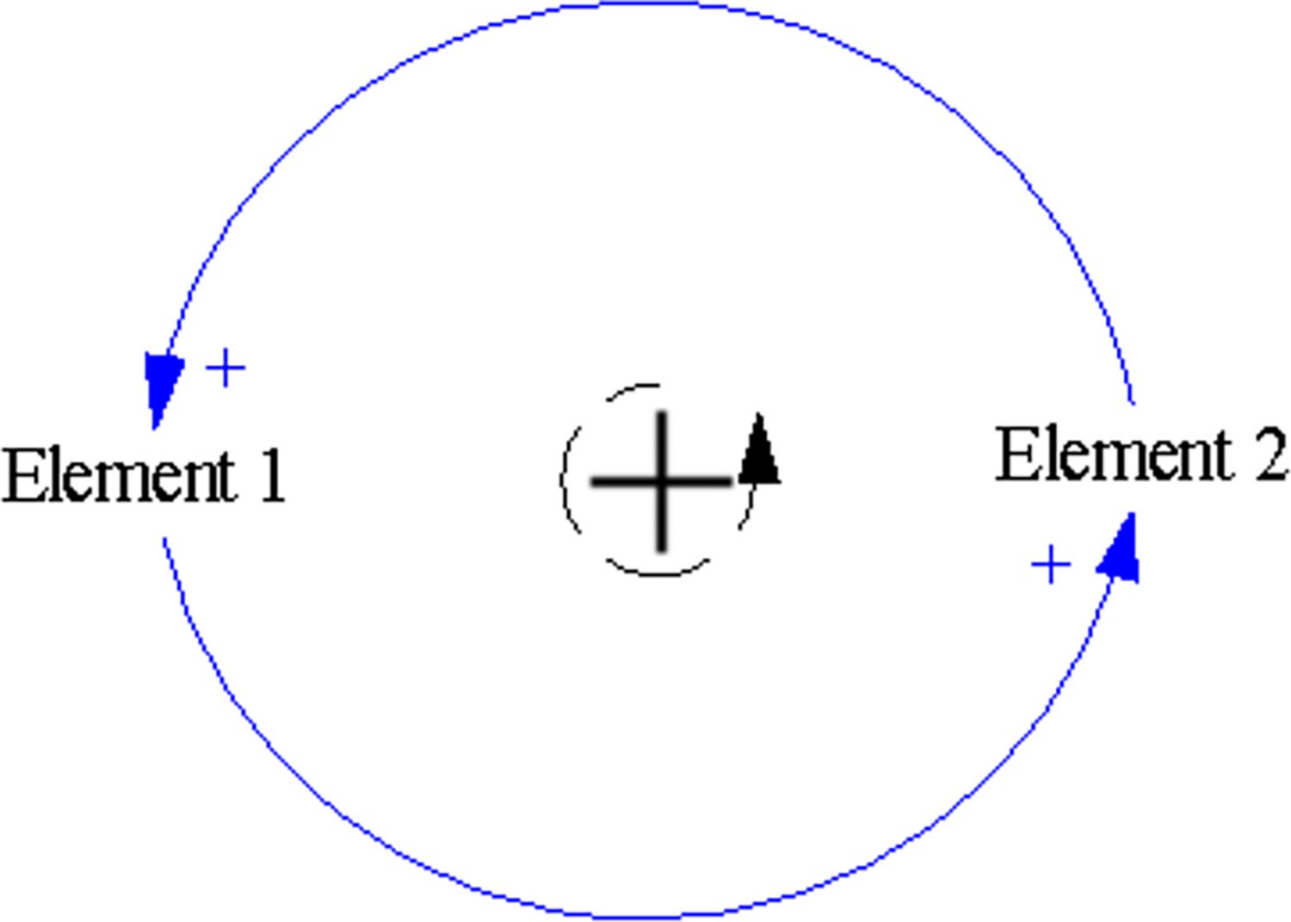
Principle of SD model.

SD is a subject integrating system science and management science. It combines system theory with computer simulation to study the structure and behavior of complex systems, reveal the internal motivations of system development and change, and analyze the effectiveness and side-effects of policies. The SD model was established by Professor Forrester at the Massachusetts Institute of Technology in the United States and is used to study enterprise inventory problems, as shown in Fig 4.

Specifically, the SD model is developed from the theories of cybernetics, reductionism, and information, which integrate theoretical analysis and simulation. The SD model searches for causal connections between components through structure-function analysis and then simulates these causal connections to depict the logical and behavioral mechanisms of the real world. By hierarchical decomposition of the system, the SD model can clarify the causal and feedback mechanisms within the system [40].

Nowadays, the SD model has been widely applied in various fields, Especially in the aspects of quantitative evaluation, dynamic simulation, scenario simulation, and path optimization, which has been proven as a mature method. The SD model is also used to study urban systems, urban agglomerations, and regional systems, which pay attention to hot issues such as economic development, environmental pollution, disaster response, knowledge networks, and the tourism industry.

Since the outbreak of the COVID-19 epidemic, many scholars have constructed SD models to predict the number of infections or simulate effective prevention and control measures. For example, Weck et al. (2020) constructed an SD model of the environment-healthcare-economy composite system and found that strict constraint policies for longer than 60 days can effectively reduce the virus infection rate but can cause serious economic losses [41]. Heidary (2022) used the SD model to analyze the destructive impact of the COVID-19 epidemic on global supply chains [42]. Hu et al. (2021) built an SD model to simulate the process of virus transmission and predicted the change in the number of infected people when the government adopted different intervention measures [43].

Resilience is a complex dynamic evolutionary process, and the understanding of evolutionary resilience further highlights the adaptability and transformational nature. Social resilience, as a specific application of resilience thinking, also exhibits dynamic changes. The change mechanism of resilience and social resilience is difficult to describe with one or several linear models. At present, scholars have attempted to construct an SD model to research resilience. For example, Frantzeskaki et al. (2017) constructed a causal feedback model in the SD model to illustrate how to enhance urban resilience [44]. Li et al. (2021) constructed an SD model to reveal the mutual feedback mechanism of various sub-dimensions of resilience [5]. Datola et al. (2022) assessed the level of resilience using an SD model [45].

**Fig 5:**
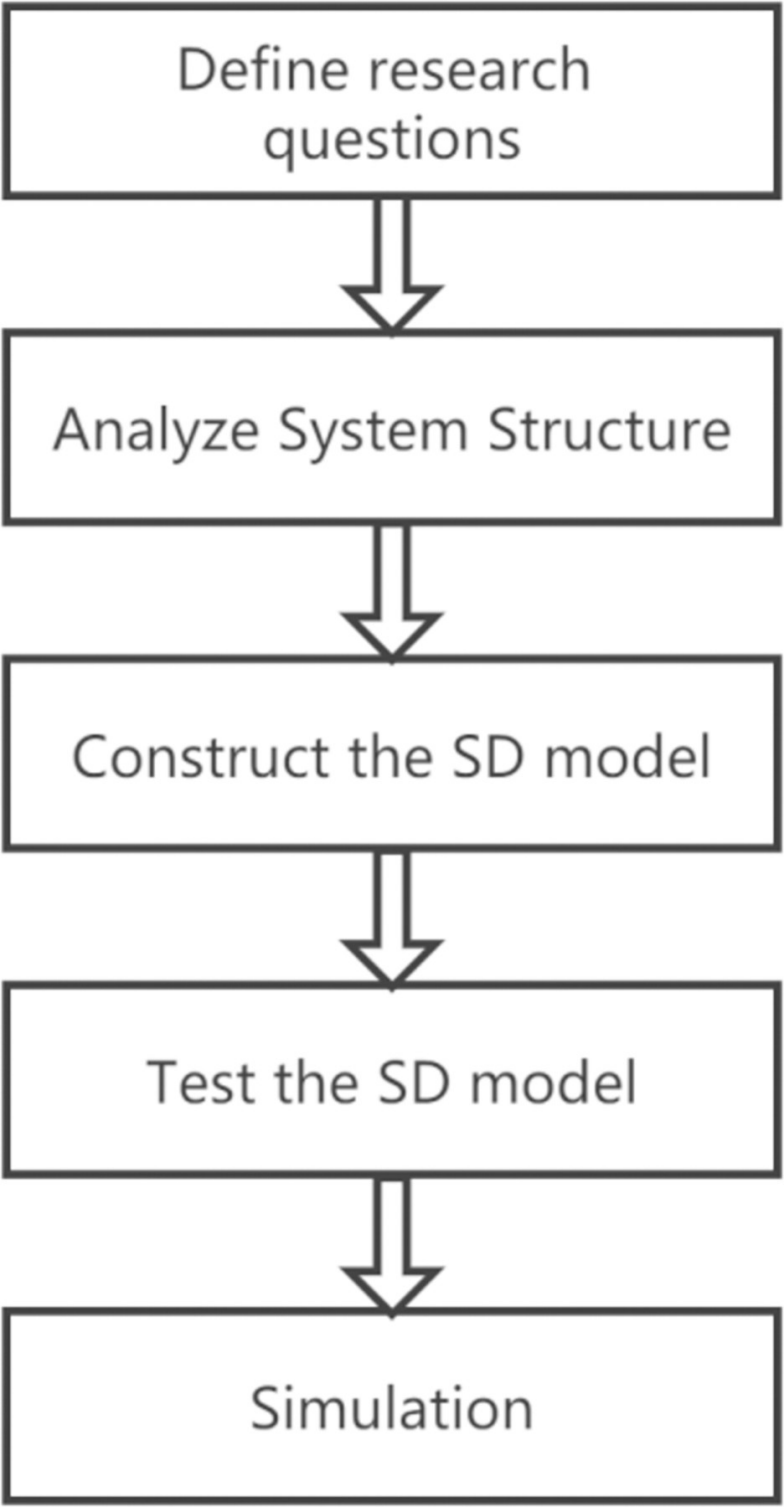
Modeling idea.

However, there is still a lack of research on using SD models to study social resilience. Compared to other resilience, such as infrastructure resilience and ecological resilience, research on social resilience is still in the assessment stage, lacking in-depth modeling and simulation research. Drawing on existing academic achievements, this article will construct an SD model and combine theoretical analysis and simulation to study the evolution process of social resilience and explore its improved measures. Following the research paradigm of the SD model, the modeling idea of this article is shown in Fig 5.

## Model development

This section first designs the structure and main variables of the model and then constructs a causal feedback model. On this basis, an SD model is further constructed.

### Model structure and variable setting

The ability of social entities to cope with risks is an important component of social resilience, so many scholars have adopted the hierarchy framework to research social entities. As proposed by Boon et al. (2016), the building of social resilience should take into account the multiple entities from micro to macro levels [46]. At present, the hierarchical division of social entities mainly includes individual, household, community, national, and global levels.

In terms of model structure, after analyzing the specific scenario of the duration of the COVID-19 epidemic, this article mainly considers three levels of social entities, namely, the central government, local governments, and the public. The central and local governments assume the primary responsibility for prevention and reconstruction, and the public is most directly affected. Firstly, the central government is at the first level from top to bottom, responsible for formulating the overall policy for combating the epidemic and allocating resources nationwide. For example, when the COVID-19 epidemic just broke out in Wuhan, the medical system of the city collapsed due to the rapid growth of the number of infected people. Subsequently, the central government allocated resources nationwide and built Leishenshan Hospital in 12 days, which greatly relieved the pressure on the medical system in Wuhan. Secondly, local governments are at the second level from top to bottom, responsible for epidemic prevention and control, social order maintenance, and resource allocation within the region. For example, during the COVID-19 epidemic, the Wuhan Municipal Government was responsible for the unified allocation and distribution of living materials, the formulation of various control policies, and the maintenance of social stability. Finally, the public is at the third level from top to bottom. After the outbreak of the COVID-19 epidemic, the daily life of the public has been greatly impacted. Many people have changed from office work to homework, and from supermarket procurement to online procurement. At the same time, many residents joined the volunteer team, cooperated with the work of communities, helped distribute living materials, nucleic acid tests, etc., and became the main force in the fight against the epidemic.

In terms of variable settings, as shown in Fig 1, the characteristics of social entities are mainly divided into structural and cognitive characteristics. Among them, cognitive characteristics have a more sustained impact on social resilience, especially characteristics such as participation, coordination, cooperation, and information exchange. Therefore, this article will focus on cognitive characteristics to set the main variables of the model. For the central government, the main consideration is epidemic awareness and emergency governance. For local governments, epidemic awareness and emergency management should also be considered. For the public, consider information acquisition and participation in cooperation.

In addition, this article mainly uses two variables to measure the level of social resilience, namely: panic degree and damage degree. The main reasons are as follows: firstly, social resilience describes the ability of the social system to maintain function and quickly recover from crises, while social loss and panic are important indicators to measure whether the entire social system is stable or has regained stability. Moreover, in the concepts of social resilience proposed by many scholars, such as Bruneau et al. (2003) and Khalili et al. (2015), social resilience is primarily aimed at reducing social disruption, losses, and chaos. Secondly, the two variables of panic degree and damage degree are closely linked. During the COVID-19 epidemic, more than once, residents have listened to rumors and rushed to supermarkets to buy goods or to pharmacies to buy large quantities of drugs, disrupting the public supply chain and hindering normal social order. Therefore, this article selects the panic degree and damage degree to represent the level of social resilience.

### Casual feedback model

**Fig 6:**
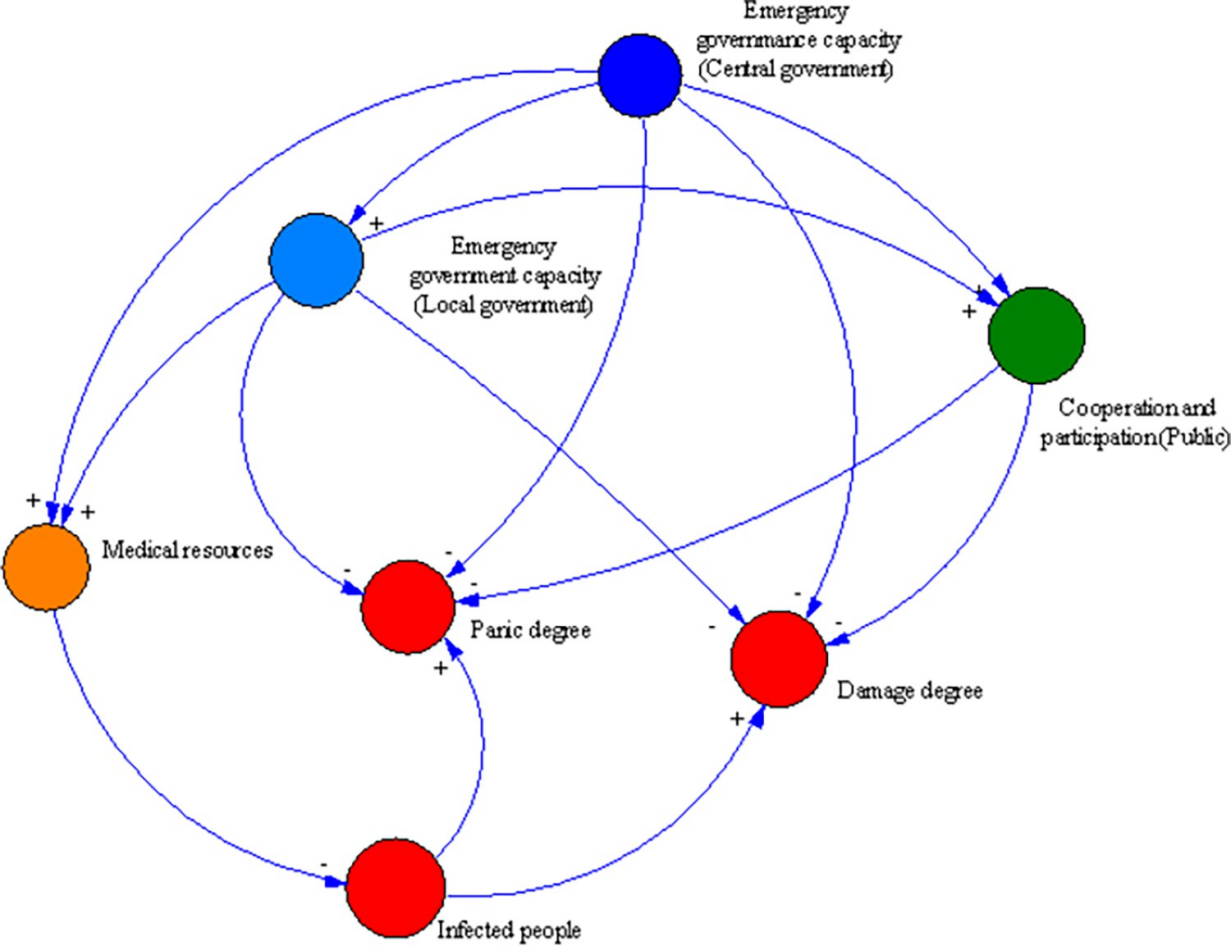
Casual feedback model.

Social resilience includes the ability of social entities to cope with risks, as well as the ability of social mechanisms and their interrelationships. The previous section elaborated on the three main social entities of this article, namely the central government, local government, and the public. This section will construct a causal feedback model to reflect the correlation between social entities. The casual feedback model can demonstrate the links between different variables. According to the hierarchy of three social entities, the casual feedback model is shown in Fig 6.

The casual feedback model consists of nodes and narrows, where the nodes represent variables and narrows represent the links between variables. A narrow marked plus sign indicates a positive relation, representing two variables changing in the same direction. On the contrary, a narrow marked minus sign indicates the negative connection, defining two variables changing in the opposite direction.

As shown in Fig 6, the red nodes represent infected people, panic degree, and damage degree, respectively. The yellow node represents medical resources. The dark blue node represents the emergency governance capacity of the central government, and the light blue represents the emergency management capacity of the local government. The green node represents the cooperation and participation of the public.

The casual feedback mechanism is as follows:

Firstly, as the spread of the virus continues to expand, more and more residents have been infected, further causing congestion in the medical system and strain on medical resources. At this point, the condition of patients who cannot receive timely treatment worsens, and the company has to shut down due to a lack of employees, resulting in an increase in social panic and damage.

Secondly, in order to ensure human health and life safety and promote the rapid restoration of normal order in the entire society, the central government has formulated a series of anti-epidemic policies and allocated resources nationwide, with a focus on assisting regions with severe epidemic situations. Local governments formulate their own anti-epidemic measures under the overall policy of the central government. During this process, the central government demonstrates emergency governance capabilities, while local governments demonstrate emergency management capabilities.

Finally, after the government continuously replenished medical resources and strengthened epidemic prevention and control measures, the speed of virus transmission was alleviated, and the growth of the number of infected individuals is also suppressed. Moreover, enterprises are gradually resuming work and production, social order is gradually restored, and the degree of social panic and damage is also decreasing.

### SD model

Based on the causal feedback model, the variables are further divided into level variables, rate variables, auxiliary variables, and constants, and an SD model is constructed. Among them, the level variable represents the variable of accumulation effect, the rate variable represents the variable of the change of accumulation effect. Auxiliary variables are usually used as intermediary variables, which are supplementary or explanatory variables to the main variable. A constant is a quantity that does not change over time and has a fixed value. The level variable, rate variable, and auxiliary variable are endogenous variables whose values are determined internally by the system, while constants are exogenous variables whose values are assigned by the external environment.

**Fig 7:**
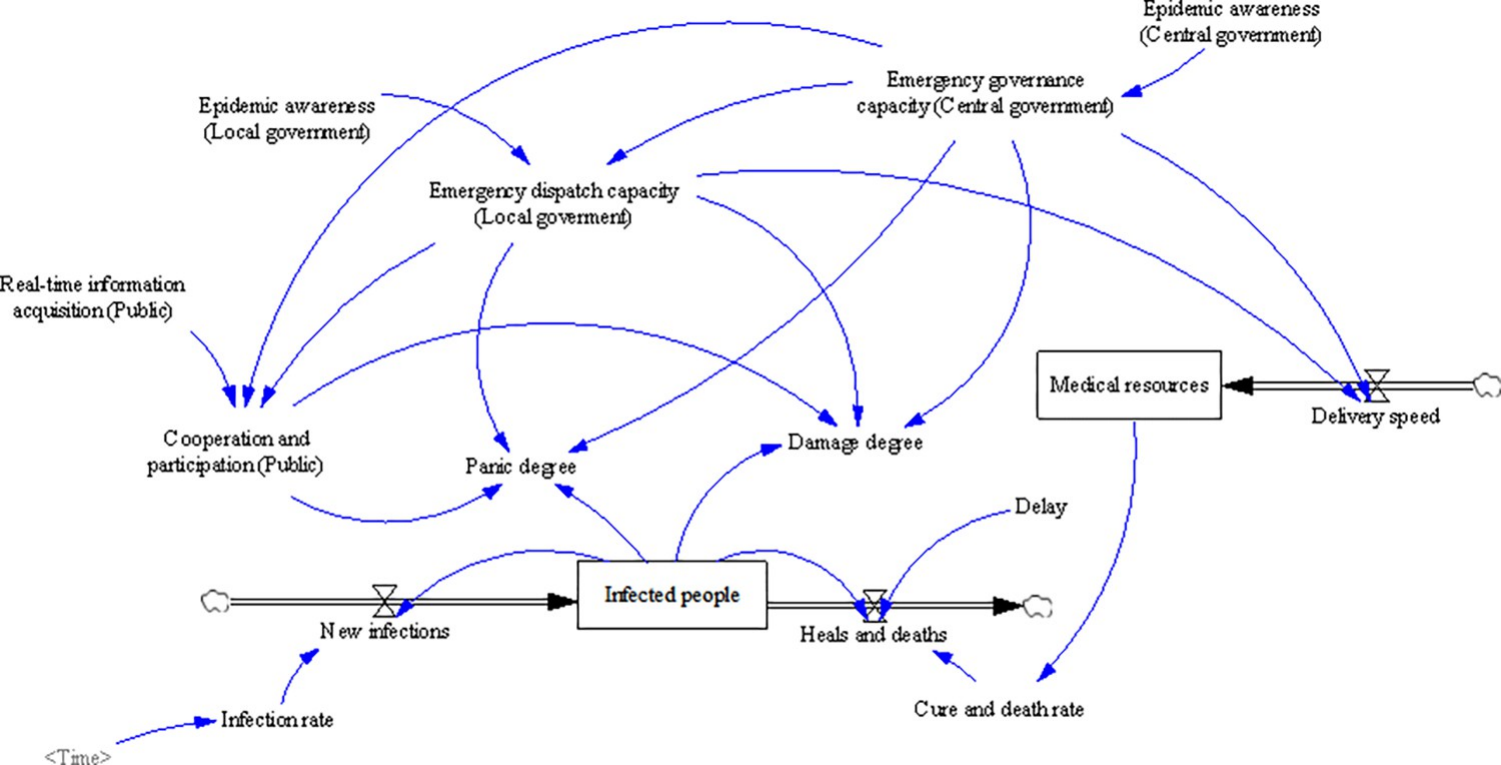
SD model.

Based on the causal feedback model shown in Fig 6, this article divides the model types as shown in Table 2, and constructs the SD model, as shown in Fig 7. The level variable is illustrated as a rectangle box, and the rate variable measures the change of the level variable, while the auxiliary and constant variables are assumed to transmit messages.

**Table 2:** Variable descriptions.

Variable type	Variable name	Description
Level variable	Infected people	Number of infected persons
	Medical resources	Quantity of necessary medical resources
Rate variable	New infections	Number of newly infected persons
	Heals and deaths	Number of healers and deaths
	Delivery speed	Delivery speed of medical resources
Auxiliary variable	Infection rate	Proportion of infected persons
	Cure rate and death rate	Proportion of healers and deaths
	Panic degree	Degree of social panic
	Damage degree	Degree of social losses
	Emergency governance capacity (Central government)	Timely and effective level of emergency governance by the central government
	Emergency management capacity (Local government)	Timely and effective level of emergency management by the local government.
	Cooperation and participation (Public)	Degree of public participation in the fight against the epidemic
Exogenous variable	Epidemic awareness (Central government)	Importance attached by the central government to epidemic prevention
	Epidemic awareness (Local government)	Importance attached by the local government to epidemic prevention
	Real-time information acquisition (Public)	Level of public access to real-time information

As shown in Fig 7, in this SD model, social resilience is represented by two variables: panic degree and damage degree, and the latter mainly refers to social losses. The variable selection of the SD model references the cognitive characteristics of social resilience. The panic degree and damage degree show a reverse trend with the level of social resilience. That is, during the COVID-19 epidemic, the higher the panic degree and damage degree, the lower the level of social resilience. On the contrary, the higher the level of social resilience. Therefore, the decrease in panic degree and damage degree indicates a strengthening of social resilience, while the increase in panic degree and damage degree indicates a weakening of social resilience. In this manner, this article can implement dynamic observation of social resilience.

This article set three exogenous variables: epidemic awareness (the central government), epidemic awareness (the local government), and real-time information acquisition (the public). The higher the epidemic awareness of the central government, the more emphasis it places on epidemic prevention and control. In this situation, the central government will quickly introduce a series of anti-epidemic policies and make every effort to allocate and ensure medical resources nationwide to curb the further spread of the virus. At the same time, as local governments are the main implementers of epidemic prevention and control policies, the higher the epidemic awareness of local governments, the faster they formulate specific epidemic prevention measures. The higher the real-time information acquisition of the public, the more conducive it is to reducing information asymmetry between the government and the public and encouraging the public to actively cooperate and participate in epidemic prevention work. In addition, all other variables are endogenous and their values are determined by the system structure and behavioral mechanisms.

## Model simulation

The previous section constructed an SD model of social resilience, which will be tested and simulated based on the Vensim platform to demonstrate the dynamic changes in social resilience during the epidemic and explore effective ways to improve it.

### Data resource and model set

This article takes Wuhan City as an example to conduct relevant research on social resilience. The COVID-19 epidemic broke out in Wuhan at the end of 2019, and then quickly spread to the whole country. Wuhan is the city most severely affected city by the epidemic in China, with a total of over 50000 confirmed cases in just four months. The research data is sourced from public reports of the National Health Commission of the People’s Republic of China (URL: http://www.nhc.gov.cn/). During the outbreak of the epidemic, the National Health Commission conducted the daily statistical analysis of the epidemic situation in Wuhan. Therefore, this article compiles all public reports from January to February 2020 and summarizes research data.

The initial time of model simulation is January 23, 2020, the final time is February 22, 2020, and the time step is one day. The simulation cycle is set as one month, as the growth of confirmed cases is the fastest during this month (from January 23 to February 22). Due to the rapid growth of confirmed cases, the local government has had to close down the entire city to curb the spread of the virus. In addition, due to the shortage of living materials and medical resources, the economic and social activities of the city have almost stagnated, and residents have also fallen into a great panic. The situation in Wuhan City makes significant sense in researching social resilience.

### Model testing

**Fig 8:**
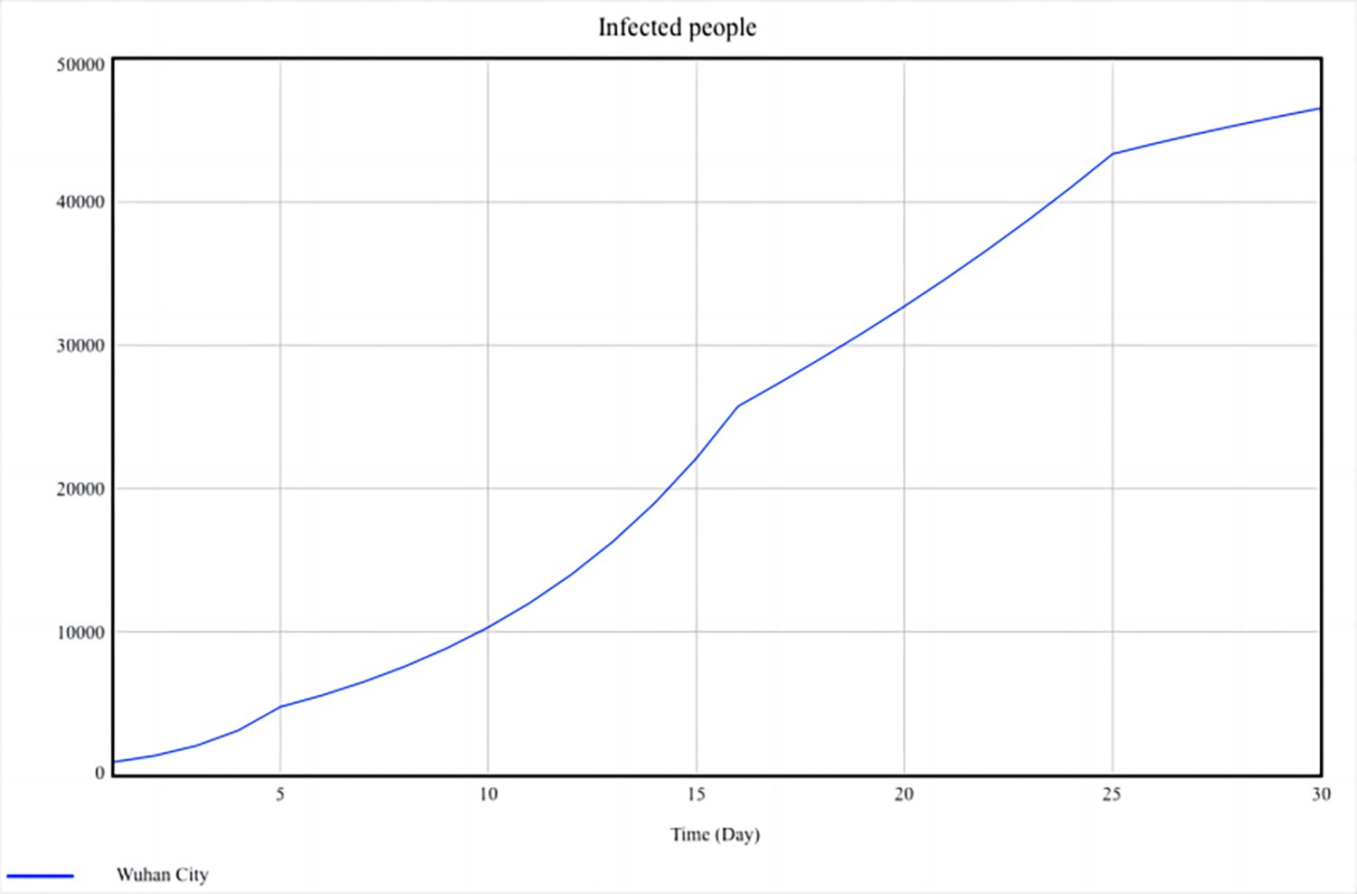
Simulation result of infected people.

**Fig 9:**
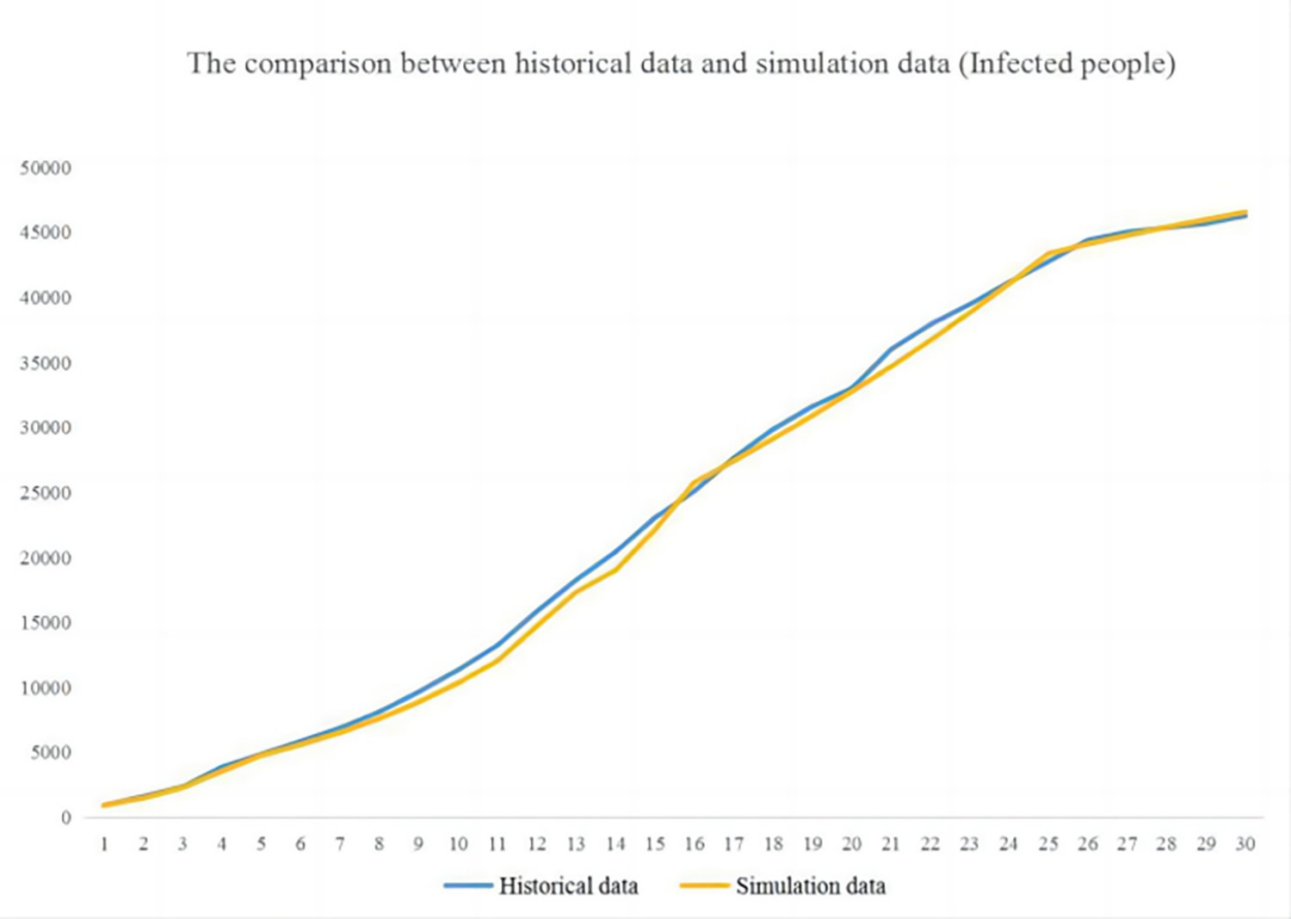
Comparison results between simulation and historical data.

The verification of the SD model is the foundation of simulation, and the most direct way is to verify whether the behavior of the model is correct, that is, whether the behavior of the model conforms to the real world. This article compares simulation data with historical data to verify the correctness of the behavior of the SD model. In the SD model shown in Fig 7, the variable “infected people” is the most important level variable, which indirectly affects the changes in other endogenous variables. Therefore, the variable “infected people” is selected as the test variable. The test results are shown in Figs 8 and 9.

Fig 8 shows a rapid increase in the number of infected people in the first half of the month (from 922 to around 22000 in just 15 days), followed by a gradual decrease in the growth rate. By the 25th day, the number of infected people had reached around 44000, and the growth rate gradually slowed down. This trend means the epidemic in Wuhan City is controlled basically, although the urban situation is still challenging.

Fig 9 shows that the simulation data of the infected people is basically consistent with the historical data, and there is no significant deviation. On days 4, 11, and 22, the deviation increased but did not exceed 10%, still within an acceptable range. Therefore, it can be considered that system dynamics models can simulate the real world.

### Simulation analysis

**Fig 10:**
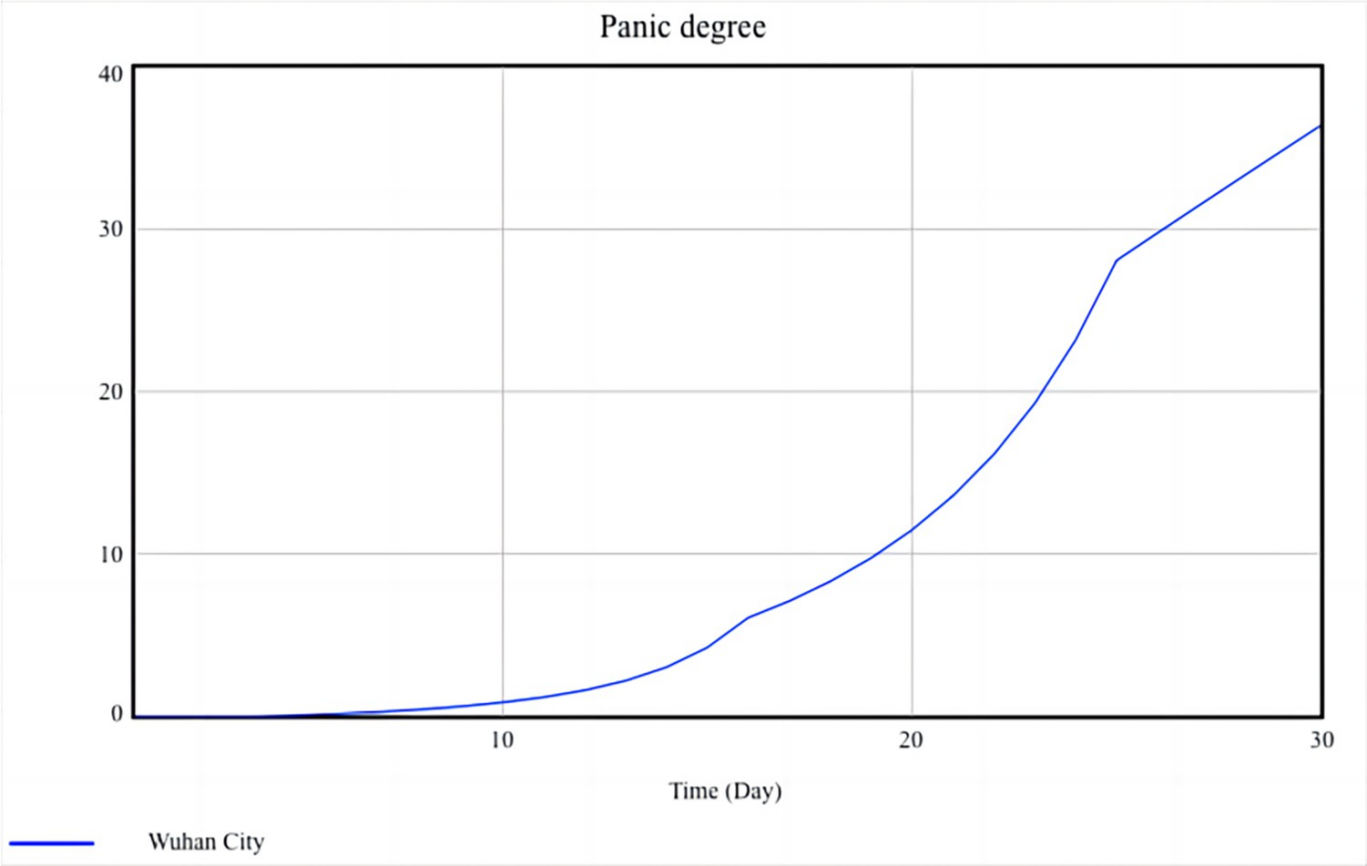
Simulation result of panic degree.

**Fig 11:**
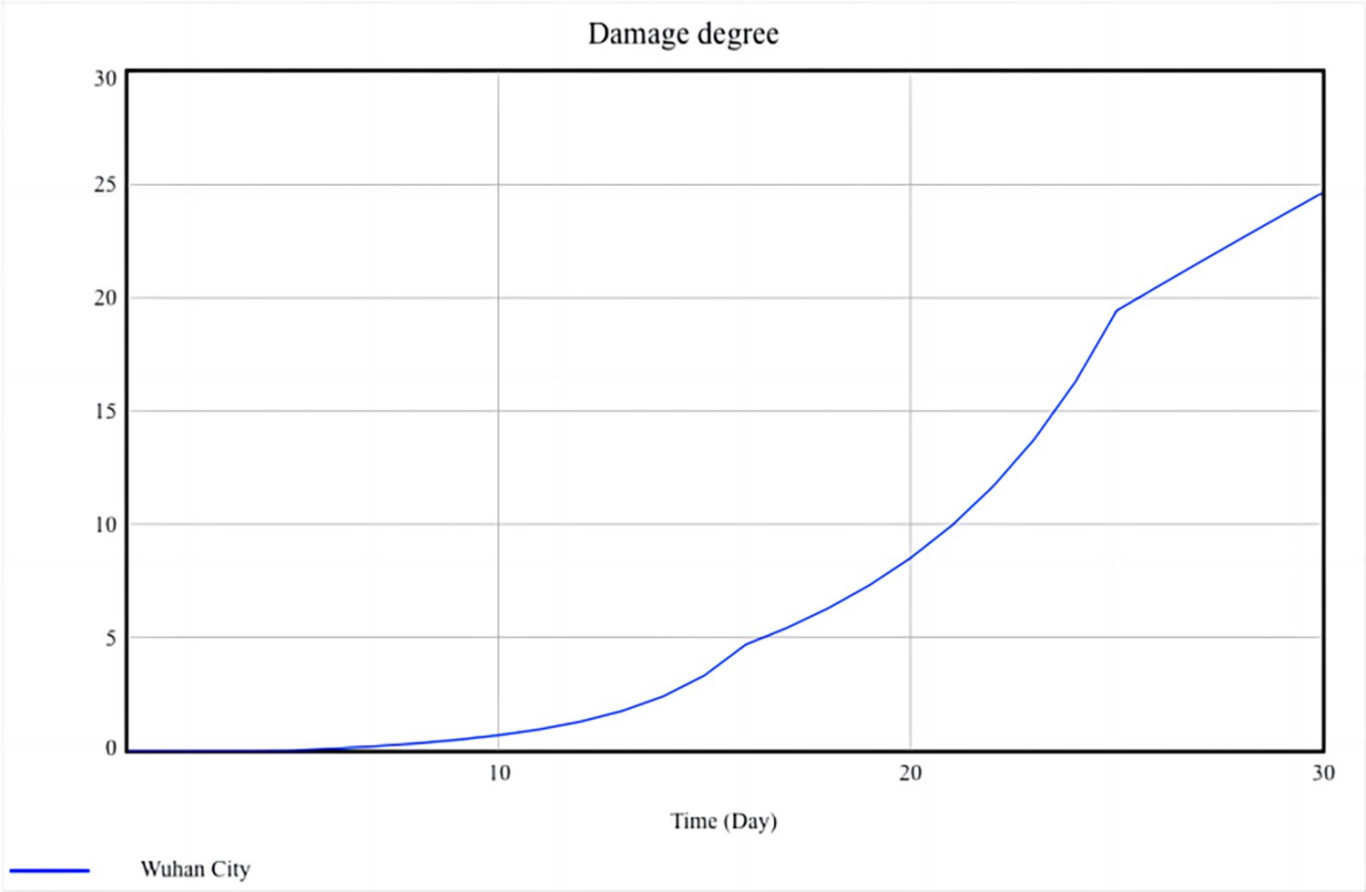
Simulation result of damage degree.

Panic degree and damage degree are used to measure the level of social resilience, and their simulation results are shown in Figs 10 and 11.

Fig 10 shows that the growth of panic degree is slow in the first half of the month, rapidly accumulating and increasing from the 15th to the 25th day, and the growth rate decreases from the 26th day onwards. Analyze the simulation results based on the actual situation in Wuhan, as follows:

In the first half of the month, although the number of infected people has been increasing every day, the public has not yet fully realized the serious impact of the epidemic on urban economic development and social order and still holds a very optimistic attitude. Most people think that they just needed to stay at home for a short time. Thus, the panic degree increases slowly during this period. On the 15th to 25th day, as more people become infected, the local government closes down the entire city. Shops close down, businesses shut down, hospitals are overcrowded, and the public begins to feel panic. The lack of living materials further deepened the panic degree. At this time, the public becomes more perceptive, and they are more likely to experience stress and anger due to losing contact with the external environment. Besides, some unreal information emerges, which spreads widely through the mass media channels and forms a network of public sentiment, and the panic degree keeps rapid growth. As the central government continues to increase its support for Wuhan, the local government has accumulated rich experience in epidemic prevention and control. The local government is increasingly timely in disclosing information and clarifying rumors that the public focuses on. people begin to calm down and reduce their overreaction. Starting from the 26th day, the number of infected people has gradually been controlled, social order has gradually recovered, and the growth rate of panic degree also slows down.

Fig 11 shows that the development trend of the damage degree is very similar to that of the panic degree: In the first half of the month, in order to curb the spread of the virus, most social activities are suspended, and businesses are also shut down. Most companies still have some cash flow to maintain operating expenses, so the growth of damage degree is relatively slow. However, as time goes on, the number of infected people has reached its peak, and companies are struggling to complete their backlog of orders due to work stoppages. At the same time, they still need to pay the rent and wages. Some companies, especially small and medium-sized enterprises, lack funds and have to lay off employees or even go bankrupt. During this period, the damage degree grows the fastest. Subsequently, the central and local governments encourage and support enterprises to resume work and production under the premise of epidemic prevention and control, and implement a series of preferential policies, such as reducing taxes and delaying the payment of employee social security funds, to help enterprises overcome difficulties. After the resumption of production, the enterprise gradually delivered orders and resumed normal operations, resulting in a slowdown in the growth of the damage degree once again.

Panic degree and damage degree are used to measure the level of social resilience during the COVID-19 epidemic. Figs 10 and 11 show the dynamic development of these two variables and also suggest the dynamic characteristics of social resilience. Due to the close correlation between panic degree and damage degree with the behavior of the public, local governments, and central government, all three of which are important social entities, it also indicates that the level of social resilience during the epidemic is significantly influenced by the behavior of social entities and social mechanisms. In addition, comparing Figs 10 and 11, it can be observed that the peak of the panic degree is higher than the damage degree. This also suggests that local governments may pay more attention to how to reduce social losses and restore the social economy during the epidemic, sometimes ignoring the public’s psychological state and failing to appease the public in a timely manner and reduce social panic.

### Scenario analysis

As mentioned earlier, the level of social resilience during the epidemic is closely related to the behavior and mechanisms of social entities. This section will explore effective ways to improve social resilience through scenario analysis. The SD model has three exogenous variables, and this article set four scenarios, accordingly, as shown in Table 3.

**Table 3:** Scenario setting.

	Epidemic awareness (Central government)	Epidemic awareness (Local government)	Real-time information acquisition (Public)
Scenario 1 (SC 1)	1	1	1
Scenario 2 (SC 2)	2	1	1
Scenario 3 (SC 3)	1	2	1
Scenario 4 (SC 4)	1	1	2

**Fig 12:**
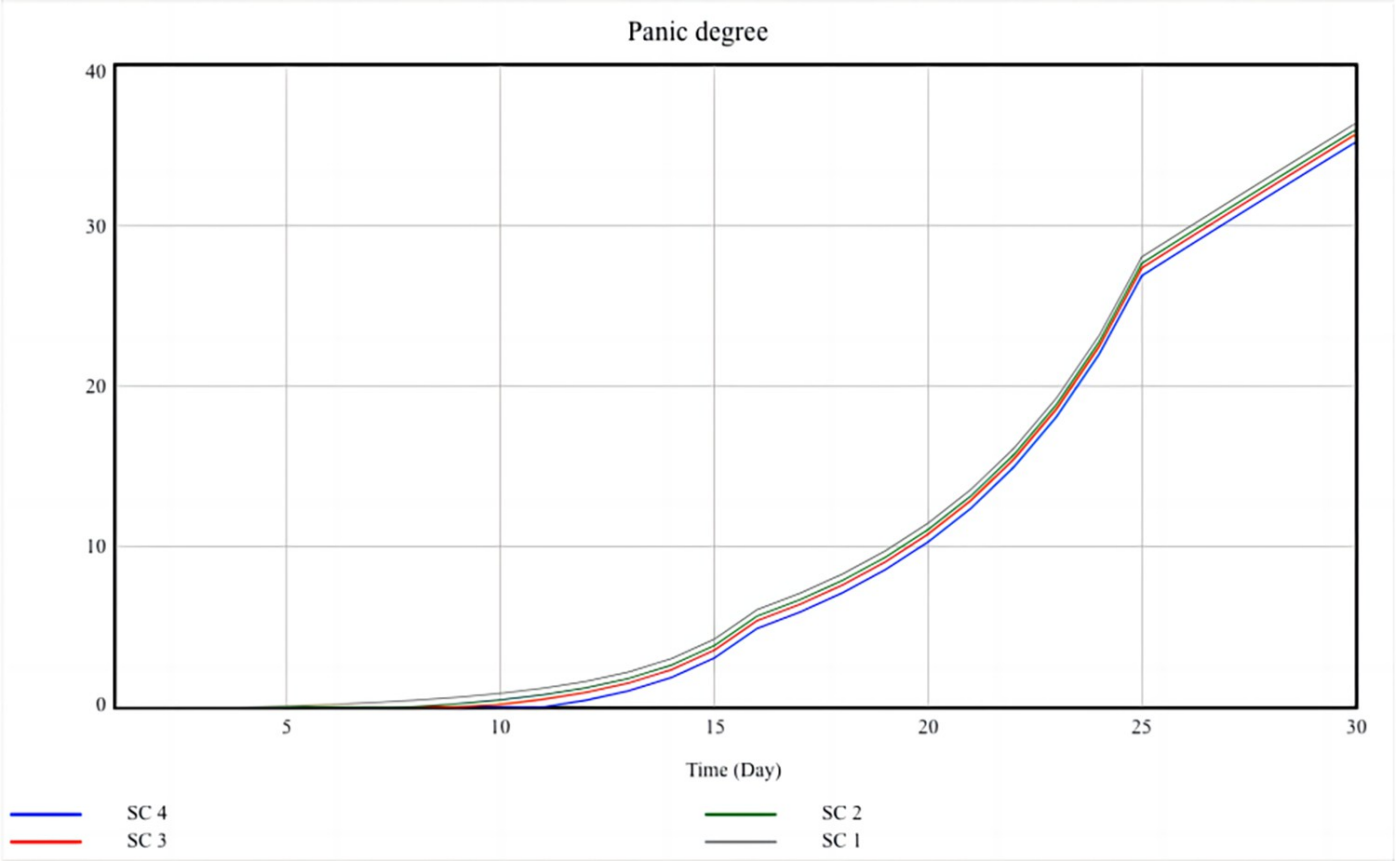
Scenario analysis result of panic degree.

**Fig 13:**
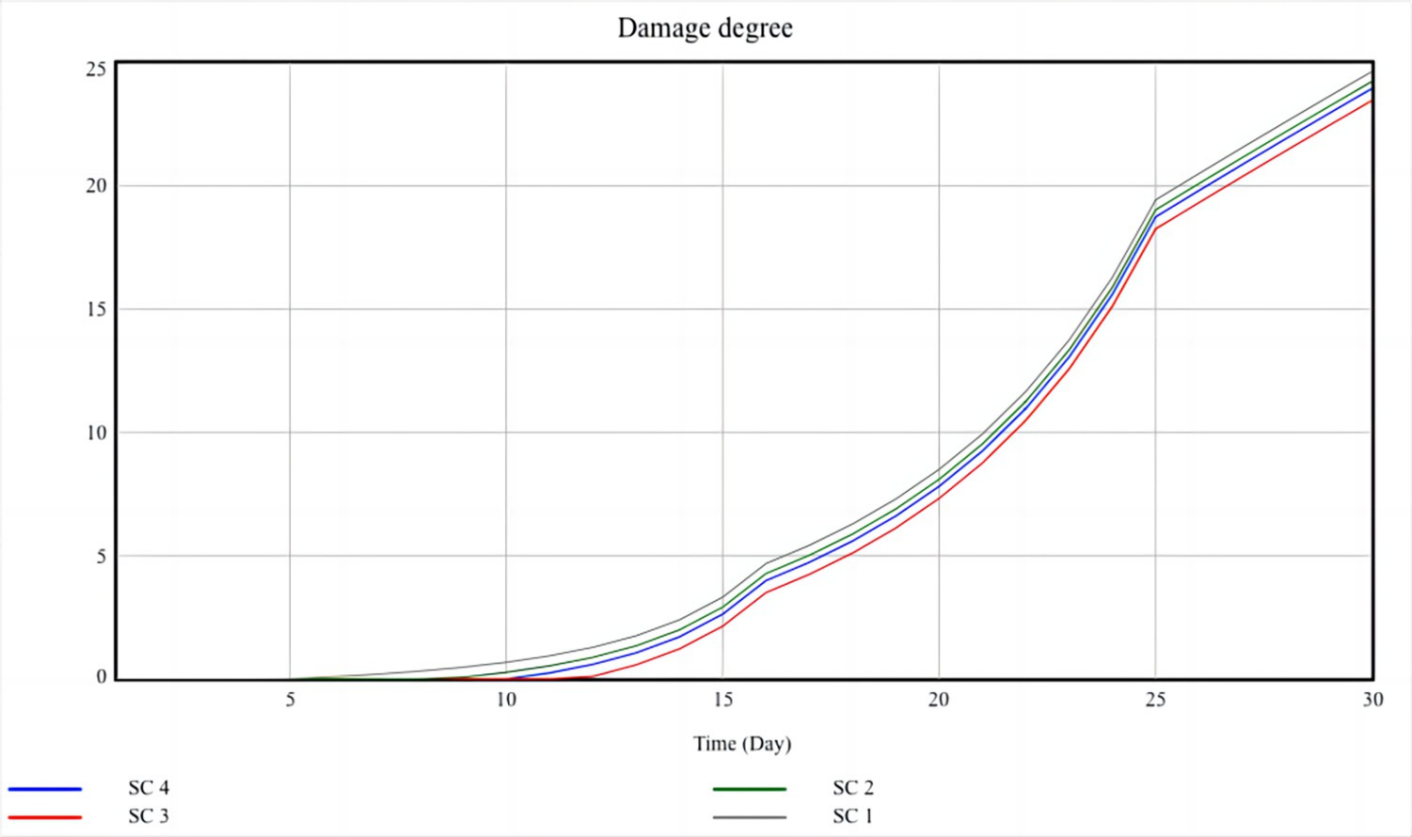
Scenario analysis result of damage degree.

As shown in Table 3, scenario 1 is the baseline scenario, and the other three scenarios are changed based on Scenario 1. For example, Scenario 2 improves the epidemic awareness of the central government, Scenario 3 improves the epidemic awareness of the local government, and Scenario 4 improves the real-time information acquisition of the public. Every scenario changes the value of only one exogenous variable at a time, and the simulation results are shown in Figs 12 and 13:

Fig 12 shows that among the four scenarios, Scenario 4 has the lowest panic degree, indicating that Scenario 4 is the optimal scenario to reduce social panic. Due to Scenario 4 changing the value of the real-time information acquisition of the public, this also indicates that the real-time information acquisition of the public is a sensitive factor in enhancing social resilience. The outbreak of the COVID-19 epidemic has completely disrupted the normal order of society. Although the central government and local governments have carried out epidemic prevention and control for the first time, due to the asymmetric information between the public and the government, it is difficult for the public to fully understand the reality situation. If the government does not disclose information related to the epidemic in a timely manner, many people will spontaneously search for information on channels such as the Internet. The Internet does less investigation of subject qualifications than traditional media like books and television, and everyone can publish and disseminate information based on social networks. Information that has not been screened for authenticity spreads rapidly through social networks, and false rumors keep cropping up and spreading through forwarding, commenting, and sharing, leading to panic behavior among the public. For example, a large number of goods and materials are stockpiled, travel information is concealed, and conflicts with community workers. On the contrary, if the public obtains more real information, they can be more proactive in cooperating with the government and community, such as accepting detection and isolation actively, thereby reducing the degree of social panic. Therefore, the real-time information acquisition of the public is a sensitive factor of the pan degree, closely related to the level of social resilience.

Fig 13 shows that among the four scenarios, Scenario 3 has the lowest damage degree, indicating that Scenario 3 is the optimal scenario to reduce social losses. Due to Scenario 3 changes the value of the epidemic awareness of local government, this also indicates that the epidemic awareness of local government is a sensitive factor in enhancing social resilience. The COVID-19 epidemic has had a serious impact on the social economy. In the first quarter of 2020, China’s GDP growth rate decreased to -6.8%, accompanied by a large number of unemployed people and the disruption of the funding chain for small and medium-sized enterprises. Therefore, on the basis of epidemic prevention and control, the government’s first priority is to quickly restore economic development. At this point, the local government plays a crucial role in economic recovery, and it formulates a series of measures to reduce the adverse effect. For example, the local government can increase residents’ consumption by issuing vouchers and supporting enterprise development through tax cuts and transfer payments. More importantly, local governments can decide on the scope and measures of epidemic prevention and control, ensuring the safety of public life while stimulating economic recovery. Therefore, the epidemic awareness of local government is a sensitive factor of the damage degree, which directly affects the level of social resilience.

## Discussion and conclusion

### Discussion

The idea of resilience originated in the field of classical physics, and its conceptual cognition has expanded from engineering resilience, and ecological resilience to evolutionary resilience. With the deepening understanding of resilience, its dynamic characteristics have gradually been revealed, and one of the representative achievements is the adaptive cycle model of resilience. Social resilience is the combination of resilience thinking and a social-ecological system, reflecting the mapping of social entity capabilities, social mechanisms, and their interrelationships. Therefore, social resilience is not a static attribute. Previous literature emphasizes the importance of conducting process-oriented characteristics or dynamic research on social resilience in the future outlook. However, research on social resilience is still in the assessment or evaluation stage. Taking the COVID-19 epidemic as an example, this article studies the dynamic change and improvement path of social resilience during the epidemic by building an SD model, which can contribute to the existing theoretical system.

Referring to existing research on defining the concept of social resilience from three perspectives: social entity capability, social mechanism capability, and both social entity and mechanism capabilities, this article selects panic degree and damage degree to measure the level of social resilience, while the remaining variables of the SD model are set around the cognitive characteristics of social resilience. The SD model is constructed and simulated on the Vensim platform. Based on the simulation results, this article obtains two important findings.

The first finding is that during the research cycle, both the panic degree and the damage degree underwent dynamic evolution under the influence of changes in social entity behavior and social mechanisms. This also indicates the dynamic evolution of social resilience and further validates the definition of social resilience. The second finding is that the real-time information acquisition of the public and the epidemic awareness of local government are sensitive factors for panic degree and damage degree, respectively. Research on social resilience places too much emphasis on the role of public organizations such as governments and communities, with less involvement in the role of the public. This article finds that if the public obtains more real-time information, it can promote the public to consciously cooperate with the epidemic prevention measures, and even actively join the volunteer team, which is conducive to reducing the panic degree. During the epidemic, local governments played a crucial role in the social order and economic recovery.

Therefore, effective measures to enhance social resilience should involve both the public and the government, including improving the public’s ability to access real-time information, increasing the timeliness of government information disclosure, and enhancing local governments’ understanding and awareness of the epidemic.

### Conclusion

Social resilience has been highlighted as one of the key factors in disaster management. Existing research on social resilience mostly focuses on assessment or evaluation, and there is still a lack of research on the dynamic characteristics and processes of social resilience, which is also a challenging issue. In view of this, this article takes the COVID-19 epidemic as an example to build a causal feedback model and a system dynamics model of social resilience based on its cognitive characteristics. Through model verification and simulation, the following conclusions are drawn:

(i) The dynamic evolution of panic degree and damage degree reveals the dynamic characteristics of social resilience, which are closely related to the behavior of social entities and social mechanisms.

(ii) The real-time information acquisition of the public and the epidemic awareness of local government are the sensitive factors for the panic degree and the damage degree, respectively. That is to say, these two factors can most significantly reduce the panic degree and damage degree, thereby enhancing the level of social resilience. Therefore, an effective way to enhance social resilience is to enhance the public’s ability to access real-time information, increase the timeliness of government information disclosure, and enhance local governments’ understanding and awareness of the epidemic.

This article still has certain limitations. For example, this article mainly considers three types of social entities: central government, local government, and the public, with less consideration given to entities such as enterprises and non-profit organizations. In addition, this article takes Wuhan as the case object, because Wuhan is the city most seriously affected by the COVID-19 epidemic in China, and less consideration is given to national and regional differences. In future research, one is to further enrich research models based on the characteristics of social resilience, and the other is to conduct multi-case validation to strengthen the universality of research models.
